# Long-term outcomes of isolated mechanical versus bioprosthetic mitral valve replacement in different age groups of propensity-matched patients

**DOI:** 10.1093/ejcts/ezae245

**Published:** 2024-06-27

**Authors:** Sorush Rokui, Byron Gottschalk, Defen Peng, Rosalind Groenewoud, Jian Ye

**Affiliations:** Division of Cardiac Surgery, St Paul’s Hospital and University of British Columbia, Vancouver, BC, Canada; Division of Cardiac Surgery, St Paul’s Hospital and University of British Columbia, Vancouver, BC, Canada; Division of Cardiac Surgery, St Paul’s Hospital and University of British Columbia, Vancouver, BC, Canada; Centre for Cardiovascular Innovation, University of British Columbia, Vancouver, BC, Canada; Division of Cardiac Surgery, St Paul’s Hospital and University of British Columbia, Vancouver, BC, Canada; Division of Cardiac Surgery, St Paul’s Hospital and University of British Columbia, Vancouver, BC, Canada

**Keywords:** Mitral valve replacement, Mechanical valve, Bioprosthetic valve, Long-term outcomes, Propensity score matching

## Abstract

**OBJECTIVES:**

Prothesis choice in isolated mitral valve replacement for patients aged 75 years or younger remains debated as most studies comparing prothesis type have included large proportions of combined operations and benefits are influenced by concomitant procedures. This study compared long-term outcomes of isolated mechanical versus bioprosthetic mitral valves in different age groups of propensity-matched populations.

**METHODS:**

This is a retrospective, multicentre, propensity-matched observational study. Baseline characteristics, operative details and long-term outcomes (mortality and freedom from surgical/transcatheter reintervention) were collected.

**RESULTS:**

Totally, 1536 isolated mitral valve replacements (806 mechanical, 730 bioprosthetic) were performed between 2000 and 2017. Over 90% of eligible patients successfully underwent propensity matching, yielding 226 each of mechanical and bioprosthetic valves in patients aged <65 years and 171 each of bioprosthetic and mechanical valves in patients aged 65–75 years with median follow-up of 13 years (maximum 20 years). In matched patients <65 years, 10-year survival was superior with mechanical valves versus bioprosthetic valves (78.2% vs 69.8%, *P* = 0.029), as was 10-year freedom from reintervention (96.2% vs 81.3%, *P* < 0.001). For matched patients between 65 and 75 years, there were no differences between mechanical and bioprosthetic valves in 10-year survival (64.6% vs 60.8%, *P* = 0.86) or 10-year freedom from reintervention (94.0% vs 97.2%, *P* = 0.23). Rates of post-operative stroke, gastrointestinal bleeding, renal failure and permanent pacemaker insertion were similar.

**CONCLUSIONS:**

In patients requiring isolated mitral valve replacement, mechanical valves confer significantly better long-term survival and freedom from reintervention for patients <65 years, while no benefit is observed at age 65–75 years compared to bioprosthetic valves.

## Graphical abstract

**Figure:**
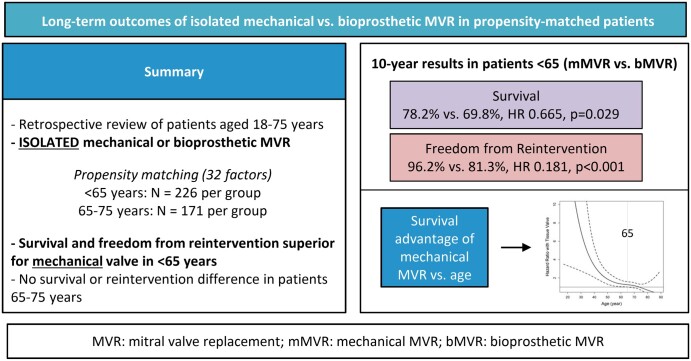

## INTRODUCTION

Mitral valve replacement (MVR) remains a common and reasonable treatment for multiple mitral valvular lesions [1]. The choice of mechanical versus bioprosthetic valves in MVR is often associated with age. Because of long-term durability, young patients often receive mechanical prostheses, whereas older patients often receive bioprostheses to avoid long-term oral anticoagulation. Current practice guidelines recommend MVR for patients with significant mitral valve disease with no option for, or failed, mitral valve repair. Class IIa recommendations prosthesis choice for MVR include bioprostheses for patients >65 years and mechanical prostheses for patients <65 years with no contraindication to anticoagulation [1]. Despite recommendations of major guidelines, contemporary practice has seen an increase in bioprosthesis use in younger patients, due in part to well-established valve-in-valve technology [2, 3].

While some contemporary studies comparing bioprosthetic versus mechanical MVR report no difference in long-term survival and/or freedom from reintervention [3–7], other studies have favoured mechanical valves, particularly in those younger than 65–70 years [8–11]. However, many of these studies have included large proportions of combined cardiac operations, wherein risks and benefits of mechanical versus bioprosthetic valves may be influenced by concomitant procedures and other heart disease [4, 6–11].

To overcome potential influences of unmatched populations, concomitant procedures and comorbid heart disease, the aim of the present study is to compare long-term outcomes of isolated mechanical versus bioprosthetic MVR—no concomitant cardiac surgery other than arrhythmia procedures—using propensity-matched populations to ascertain the role of prosthesis choice in long-term surgical outcomes without the influence of concomitant procedures.

## PATIENTS AND METHODS

### Ethical statement

The University of British Columbia institutional review board approved this research (reference: H17-03009, initial approval 11 November 2011). Patient’s informed consent was waived given the retrospective study design.

### Study design

Data were collected from patients who underwent isolated MVR at 5 hospitals across the province of British Columbia, Canada, between January 2000 and December 2017, to evaluate effects of mechanical versus bioprosthetic valves on mortality, reintervention and peri- and post-operative complications. Data were collected from Cardiac Services British Columbia’s central database, a province-wide registry where data submission is mandatory. The registry contains prospectively collected data from every patient who undergoes any cardiac surgery or interventions. Demographics, socioeconomic factors, chronic conditions, operative characteristics, early morbidity and mortality, long-term mortality and surgical or transcatheter (valve in valve) reintervention were included.

### Study population

Every consecutive patient with MVR with mechanical or bioprosthetic valves between January 2000 and December 2017 was identified from the provincial registry with prospective data collection. Patients were excluded if aged <18 years, >75 years or had any form of concomitant cardiac surgery except left atrial appendage amputation, surgical pulmonary vein ablation or Maze procedures. Consequently, 1536 isolated MVRs (806 mechanical, 730 bioprosthetic) were identified. We stratified patients by age: categories were 18–64 years and 65–75 years at time of surgery, reflecting current practice guidelines as well as local practice pattern. Regarding propensity score matching, 226/252 patients aged <65 years with bioprosthetic MVR were matched, 171/184 patients aged 65–75 years with mechanical MVR were matched and 51.7% of the total study population was successfully matched (Supplementary Material, Table S1). This yielded 452 patients aged <65 years (226 patient pairs) and 342 patients aged 65–75 years (171 patient pairs). Previous cardiac surgery was not an exclusion criterion. Regarding anticoagulation following MVR, our regional general practice includes lifelong anticoagulation for patients receiving mechanical MVR, and a minimal 3 months of anticoagulation for patients receiving bioprosthetic MVR if there is no other indication for anticoagulation, provided there are no contraindications to anticoagulation.

### Outcomes

Primary end-points were mortality and freedom from reintervention. Mortality data were obtained via British Columbia Vital Statistics. Reintervention rates were collected from the provincial registry database. Secondary end-points included in-hospital complications such as stroke, gastrointestinal bleed, renal failure and arrhythmia necessitating permanent pacemaker insertion.

### Statistical analysis

#### Missing data

Missing data at baseline were infrequent (<1% for most variables); however, hemoglobin, creatinine and left ventricular ejection fraction were missing in 11.2%, 5.8% and 4.5% of patients, respectively. To minimize bias and maximize use of available information in statistical analysis, imputations were performed with the multiple imputation approach using multivariate normal distribution assuming ‘missing at random’ and number of imputations to be performed was specified as 5 for higher accuracy.

#### Statistical analysis before propensity score matching

Continuous variables were reported as mean ± standard deviation or median and interquartile range (P25, P75) and examined with *Student's t-test* or Wilcoxon rank sum test, except for survivals, which were reported as mean ± standard error or confidence interval. Categorical variables were presented as frequency (percentage) and examined with Chi-squared test or Fisher’s exact test between patients with mechanical valve and bioprosthetic valve. The relationship between short-term mortality and valve type was analyzed using logistic regression model. Kaplan–Meier methods were used to examine survival by the categorical factors studied. Categorical predictors of outcomes were individually tested for equality of survival with log-rank test. Relationships between valve types and long-term mortality were also explored with Cox proportional hazards regression model. The proportional hazards assumption of Cox regressions was tested based on Schoenfeld residuals. Sub-distribution hazard model considering death as competing risks was conducted to explore how valve type affects re-do isolated MVR [12].

#### Propensity score matching

A propensity-matched comparison was used to control for potentially confounding variables because of significant differences in baseline characteristics and risk factors between mechanical and bioprosthetic valve patients. A logistic regression based on 32 demographic and risk factors was used to generate a propensity score for each patient. Pairs of patients with mechanical or bioprosthetic valves were derived using greedy one-to-one matching with an absolute difference between the propensity scores of 0.20.

After propensity score matching, McNemar’s test or conditional logistic model was used for analysis of categorical variables, paired t-test for normally distributed continuous variables and Wilcoxon signed rank sum test for non-normally distributed continuous variables. The quality of matching was also assessed using standardized mean difference [13]. A robust variance estimator was used to account for clustering within matched sets when using logistic regression model or Cox proportional hazards model to regress short-term/long-term outcomes on prosthetic valve types, or sub-distribution hazard model considering death as competing risks to explore how valves type affects re-do isolated MVR. Thirty-day mortalities were excluded from Cox proportional hazards modelling on long-term mortality.

In addition to propensity-score matching, inverse probability of treatment weighting analysis was performed in order to detect any potential bias in the propensity-score matched comparison [14].

The conventional 5% level of significance was used as a nominal reporting level. All tests were 2-sided. All statistical analyses were performed using SAS software version 9.4 (SAS Institute, Cary, North Carolina) and R software version 4.3.2 (R Foundation for Statistical Computing, Vienna, Austria).

## RESULTS

### Patients

During the study period, 1536 patients aged 18–75 received isolated MVR representing 47% of all MVR operations performed: 874 patients aged <65 years and 662 patients aged 65–75 years. Of these, 806 received mechanical prostheses and 730 patients received bioprostheses (Supplementary Material, Table S1). Median follow-up time was 13.0 (7.5, 17.1) years (mechanical: 14.4 [8.7, 17.9] years, bioprosthetic: 11.3 [6.8, 15.8] years). Mean follow-up time was 9.4 ± 5.8 years (mechanical: 10.3 ± 6.1 years, bioprosthetic: 8.5 ± 5.4 years). Total follow-up time was 3214.8 patient-years (mechanical: 1504.8 patient-years, bioprosthetic: 1710.0 patient-years). Follow-up for the primary end-points was achieved in 100% of study patients as reporting any death or cardiac intervention to British Columbia Vital Statistics or Cardiac Registry are mandatory, respectively. The dominant pathology types included rheumatic (35%) and degenerative (28%) valvular diseases. Other less common pathologies included prosthetic valve dysfunction, endocarditis and ischaemic mitral disease. Valvular prostheses commonly used for replacement included Medtronic porcine (18.0%), Edwards bovine (14.3%) and St Jude Medical porcine (7.2%) bioprostheses; mechanical prostheses included St Jude Medical (29.1%), On-X (10.3%) and Carbomedics (10.3%). At baseline, patients receiving bioprostheses were older (64.6 ± 11.3 vs 55.5 ± 11.2 years, *P* < 0.001), presented with more severe symptoms (26.4% vs 15.3% New York Heart Association (NYHA) IV symptoms, *P* < 0.001) and had more comorbidities (including hypertension, peripheral vascular disease, renal failure, gastrointestinal bleed, malignancy history and myocardial infarction history) compared with those receiving mechanical prostheses (Supplementary Material, Tables S2 and S3). Using 32 demographic and risk factors summarized in Table 1, propensity score matching yielded 397 patient pairs: 226 patients each of bioprosthetic and mechanical MVR in the <65 years age group and 171 patients each of bioprosthetic and mechanical MVR in the 65–75 years age group (Tables 1 and 2, respectively). Following propensity score matching, no significant differences were observed in 32 baseline characteristics between bioprosthetic and mechanical MVR in propensity-matched data (Tables 1 and 2).

**Table 1:** Baseline characteristics of propensity-matched cohort (aged <65 years)

Variable	All *n* = 452	Bioprosthetic *n* = 226	Mechanical *n* = 226	*P*-value	SMD
Surgery age (years)	52.1 ± 10.4	52.6 ± 11.4	51.7 ± 9.3	0.38	−8.3
Sex (Male)	209 (46.2)	110 (48.7)	99 (43.8)	0.30	−9.8
BMI (kg/m^2^)	25.8 ± 5.9	25.6 ± 5.8	26.0 ± 6.1	0.48	6.7
Creatinine (μmol/l)	110.9 ± 105.7	110.1 ± 111.0	111.7 ± 100.4	0.87	1.6
Hemoglobin (g/l)	123.7 ± 22.3	123.9 ± 23.2	123.5 ± 21.3	0.86	−1.7
CCS class					10.3
None	378 (83.6)	187 (82.7)	191 (84.5)		
Class 1 or 2	38 (8.4)	22 (9.7)	16 (7.1)	0.29	
Class 3 or 4	36 (8.0)	17 (7.5)	19 (8.4)	0.71	
NYHA class					9.5
None or Class I	44 (9.7)	22 (9.7)	22 (9.7)		
Class II	116 (25.7)	60 (26.5)	56 (24.8)	0.81	
Class III	176 (38.9)	89 (39.4)	87 (38.5)	0.97	
Class IV	116 (25.7)	55 (24.3)	61 (27.0)	0.58	
Ejection fraction (≤50%)	158 (35.0)	79 (35.0)	79 (35.0)	0.99	0.0
Emergency or priority I for surgery	105 (23.2)	53 (23.5)	52 (23.0)	0.90	−1.1
Prior MI	56 (12.4)	27 (11.9)	29 (12.8)	0.76	2.7
CHF	347 (76.8)	174 (77.0)	173 (76.5)	0.91	−1.1
Active endocarditis	65 (14.4)	33 (14.6)	32 (14.2)	0.88	−1.3
Hypertension	173 (38.3)	87 (38.5)	86 (38.1)	0.92	−0.9
Pulmonary hypertension	252 (55.8)	125 (55.3)	127 (56.2)	0.85	1.8
Family history of CAD	70 (15.5)	36 (15.9)	34 (15.0)	0.79	−2.5
Dyslipidaemia	124 (27.4)	63 (27.9)	61 (27.0)	0.83	−2.0
Pre-op arrhythmia	115 (25.4)	61 (27.0)	54 (23.9)	0.44	−7.1
Pre-op IABP	4 (0.9)	1 (0.4)	3 (1.3)	0.32	9.5
PVD	28 (6.2)	14 (6.2)	14 (6.2)	0.99	0.0
COPD	127 (28.1)	60 (26.5)	67 (29.6)	0.46	6.9
Pre-op ventilation/intubation	28 (6.2)	14 (6.2)	14 (6.2)	0.99	0.0
Stroke/TIA	73 (16.2)	40 (17.7)	33 (14.6)	0.37	−8.4
Renal failure—acute	28 (6.2)	14 (6.2)	14 (6.2)	0.99	0.0
Renal failure—chronic	28 (6.2)	14 (6.2)	14 (6.2)	0.99	0.0
Dialysis	22 (4.9)	10 (4.4)	12 (5.3)	0.64	4.1
Diabetes	51 (11.3)	28 (12.4)	23 (10.2)	0.48	−7.0
GI bleed history	35 (7.7)	18 (8.0)	17 (7.5)	0.85	−1.7
Malignant disease controlled < 5 years	9 (2.0)	6 (2.7)	3 (1.3)	0.26	−9.5
Smoking history—smoker or d/c < 1 mo	81 (17.9)	40 (17.7)	41 (18.1)	0.90	1.2
History of drug abuse	36 (8.0)	18 (8.0)	18 (8.0)	0.99	0.0
PCI	26 (5.8)	11 (4.9)	15 (6.6)	0.43	7.6
Previous open heart surgery	53 (11.7)	27 (11.9)	26 (11.5)	0.88	−1.4

Values are shown as mean ± SD or *n* (%). Baseline characteristics for unmatched data are presented in Supplementary Material, Table S4.

BMI: body mass index; CAD: coronary artery disease; CCS: Canadian Cardiovascular Society; CHF: congestive heart failure; COPD: chronic obstructive pulmonary disease; GI: gastrointestinal; IABP: intra-aortic balloon pump; MI: myocardial infarction; NYHA: New York Heart Association; PCI: percutaneous coronary intervention; PVD: peripheral vascular disease; SMD: standardized mean difference; TIA: transient ischaemic attack.

**Table 2:** Baseline characteristics of propensity-matched cohort (aged 65–75 years)

Variable	All *n* = 342	Bioprosthetic *n* = 171	Mechanical *n* = 171	*P*-value	SMD
Surgery age (years)	69.0 ± 2.9	69.0 ± 3.0	68.9 ± 2.7	0.66	−4.7
Sex (Male)	133 (38.9)	67 (39.2)	66 (38.6)	0.92	−1.2
BMI (kg/m^2^)	26.5 ± 5.4	26.5 ± 5.6	26.5 ± 5.2	0.97	0.4
Creatinine (μmol/l)	101.6 ± 69.8	102.2 ± 85.5	101.0 ± 49.6	0.87	−1.7
Hemoglobin (g/l)	131.1 ± 15.8	131.0 ± 15.9	131.2 ± 15.9	0.86	1.7
CCS class					2.3
None	287 (83.9)	143 (83.6)	144 (84.2)		
Class 1 or 2	40 (11.7)	22 (12.9)	18 (10.5)	0.55	
Class 3 or 4	15 (4.4)	6 (3.5)	9 (5.3)	0.42	
NYHA class					6.3
None or Class I	24 (7.0)	11 (6.4)	13 (7.6)		
Class II	69 (20.2)	36 (21.1)	33 (19.3)	0.61	
Class III	191 (55.8)	95 (55.6)	96 (56.1)	0.74	
Class IV	58 (17.0)	29 (17.0)	29 (17.0)	0.76	
Ejection fraction (≤50%)	137 (40.1)	67 (39.2)	70 (40.9)	0.74	−3.6
Emergency or priority I for surgery	52 (15.2)	27 (15.8)	25 (14.6)	0.77	−3.3
Prior MI	39 (11.4)	20 (11.7)	19 (11.1)	0.86	−1.8
CHF	289 (84.5)	147 (86.0)	142 (83.0)	0.43	−8.1
Active endocarditis	8 (2.3)	4 (2.3)	4 (2.3)	0.99	0.0
Hypertension	191 (55.8)	92 (53.8)	99 (57.9)	0.44	8.3
Pulmonary hypertension	207 (60.5)	104 (60.8)	103 (60.2)	0.90	−1.2
Family history of CAD	43 (12.6)	22 (12.9)	21 (12.3)	0.87	−1.8
Dyslipidaemia	135 (39.5)	68 (39.8)	67 (39.2)	0.91	−1.2
Pre-op arrhythmia	186 (54.4)	94 (55)	92 (53.8)	0.83	−2.3
Pre-op IABP	2 (0.6)	1 (0.6)	1 (0.6)	0.99	0.0
PVD	11 (3.2)	4 (2.3)	7 (4.1)	0.37	10.0
COPD	67 (19.6)	33 (19.3)	34 (19.9)	0.89	1.5
Pre-op ventilation/intubation	2 (0.6)	1 (0.6)	1 (0.6)	0.99	0.0
Stroke/TIA	59 (17.3)	25 (14.6)	34 (19.9)	0.21	14.0
Renal failure—acute	10 (2.9)	5 (2.9)	5 (2.9)	0.99	0.0
Renal failure—chronic	15 (4.4)	5 (2.9)	10 (5.8)	0.17	14.3
Dialysis	6 (1.8)	3 (1.8)	3 (1.8)	0.99	0.0
Diabetes	56 (16.4)	27 (15.8)	29 (17.0)	0.79	3.2
GI bleed history	9 (2.6)	4 (2.3)	5 (2.9)	0.74	3.7
Malignant disease controlled < 5 years	18 (5.3)	8 (4.7)	10 (5.8)	0.62	5.2
Smoking history—smoker or d/c < 1 mo	16 (4.7)	7 (4.1)	9 (5.3)	0.59	5.5
History of drug abuse	0 (0.0)	0 (0.0)	0 (0.0)		0.0
PCI	26 (7.6)	13 (7.6)	13 (7.6)	0.99	0.0
Previous open heart surgery	40 (11.7)	21 (12.3)	19 (11.1)	0.72	−3.6

Values are shown as mean ± SD or *n* (%). Baseline characteristics for unmatched data are presented in Supplementary Material, Table S5.

BMI: body mass index; CAD: coronary artery disease; CCS: Canadian Cardiovascular Society; CHF: congestive heart failure; COPD: chronic obstructive pulmonary disease; GI: gastrointestinal; IABP: intra-aortic balloon pump; MI: myocardial infarction; NYHA: New York Heart Association; PCI: percutaneous coronary intervention; PVD: peripheral vascular disease; SMD: standardized mean difference; TIA: transient ischaemic attack.

### Early outcomes

Outcomes of patients aged <65 after matching are summarized in Table 3. Comparing mechanical and bioprosthetic MVR, cardiopulmonary bypass and aortic cross-clamping times were similar. In-hospital post-operative complications including stroke (1.8 vs 0.9%, *P* = 0.41), gastrointestinal bleeding (0.9% in both valve types) and permanent pacemaker insertion (1.8% vs 1.3%, *P* = 0.71) were also similar between valve types. Acute renal failure requiring dialysis occurred less frequently in patients receiving a mechanical valve (2.7% vs 8.0%, *P* = 0.011). Importantly, 30-day mortality was similar between groups (4.0% vs 3.1%, *P* = 0.62).

**Table 3:** In-hospital and out-of-hospital outcomes of propensity-matched cohort (aged < 65 years)

Variable	All *n* = 452	Tissue *n* = 226	Mechanical *n* = 226	*P*-value
Pump time (min)	123.0 (94.0, 166.0)	123.0 (93.0, 160.0)	123.0 (95.0, 171.0)	0.29
Cross clamp time (min)	94.0 (72.0, 131.0)	95.0 (71.0, 130.0)	91.5 (72.0, 134.5)	0.37
Creatinine-post (μmol/l)	97.6 ± 85.2	94.4 ± 77.0	100.8 ± 92.9	0.45
Prosthetic valve endocarditis	0 (0.0)	0 (0.0)	0 (0.0)	
Insertion of permanent pacemaker	7 (1.5)	3 (1.3)	4 (1.8)	0.71
Post-op haemorrhage/tamponade	10 (2.2)	5 (2.2)	5 (2.2)	0.99
Arrhythmia—cardiac arrest	5 (1.1)	3 (1.3)	2 (0.9)	0.65
Arrhythmia—atrial	169 (37.4)	79 (35.0)	90 (39.8)	0.27
Arrhythmia—heart block	23 (5.1)	12 (5.3)	11 (4.9)	0.83
Valvular thromboembolism/thrombosis	0 (0.0)	0 (0.0)	0 (0.0)	
Inotropes > 24 h	31 (6.9)	13 (5.8)	18 (8.0)	0.35
Stroke	6 (1.3)	2 (0.9)	4 (1.8)	0.41
Acute renal failure requiring dialysis	24 (5.3)	18 (8.0)	6 (2.7)	0.011
Acute failure without dialysis	16 (3.5)	6 (2.7)	10 (4.4)	0.32
Gastrointestinal bleed	4 (0.9)	2 (0.9)	2 (0.9)	0.99
Prolonged ventilation	17 (3.8)	8 (3.5)	9 (4.0)	0.81
30-day mortality	16 (3.5)	7 (3.1)	9 (4.0)	0.62
One-year mortality	34 (7.5)	17 (7.5)	17 (7.5)	0.99
Mortality during follow-up	131	71	60	
Reintervention during follow-up	39	32	7	

Values are shown as mean ± SD or median (interquartile) or *n* (%). Calculation based on complete observations. Summaries for unmatched data are presented in Supplementary Material, Table S6.

Outcomes of patients aged 65–75 after matching are summarized in Table 4. Comparing mechanical and bioprosthetic MVR, cardiopulmonary bypass and aortic cross-clamping times were similar. In-hospital post-operative complications including stroke (3.5% vs 1.2%, *P* = 0.16), gastrointestinal bleeding (1.2% in both valve types), permanent pacemaker insertion (1.2% vs 1.8%, *P* = 0.65) and acute renal failure requiring dialysis (2.3% vs 4.1%, *P* = 0.37) were also similar between valve types. Again, 30-day mortality was similar between groups (5.8% vs 2.3%, *P* = 0.12).

**Table 4:** In-hospital and out-of-hospital outcomes of propensity-matched cohort (aged 65–75 years)

Variable	All *n* = 342	Tissue *n* = 171	Mechanical *n* = 171	*P*-value
Pump time (min)	128.0 (99.0, 168.0)	130.0 (100.0, 169.0)	125.0 (96.0, 167.0)	0.67
Cross clamp time (min)	97.5 (76.0, 134.0)	100.0 (79.0, 134.0)	94.5 (73.0, 132.0)	0.72
Creatinine-post (μmol/l)	97.4 ± 85.9	94.6 ± 71.3	100.5 ± 99.3	0.56
Prosthetic valve endocarditis	1 (0.3)	1 (0.6)	0 (0.0)	
Insertion of permanent pacemaker	5 (1.5)	3 (1.8)	2 (1.2)	0.65
Post-op haemorrhage/tamponade	12 (3.5)	6 (3.5)	6 (3.5)	0.99
Arrhythmia—cardiac arrest	4 (1.2)	2 (1.2)	2 (1.2)	0.99
Arrhythmia—atrial	153 (44.7)	81 (47.4)	72 (42.1)	0.35
Arrhythmia—heart block	18 (5.3)	9 (5.3)	9 (5.3)	0.99
Valvular thromboembolism/thrombosis	0 (0.0)	0 (0.0)	0 (0.0)	
Inotropes > 24 h	18 (5.3)	6 (3.5)	12 (7.0)	0.13
Stroke	8 (2.3)	2 (1.2)	6 (3.5)	0.16
Acute renal failure requiring dialysis	11 (3.2)	7 (4.1)	4 (2.3)	0.37
Acute failure without dialysis	16 (4.7)	8 (4.7)	8 (4.7)	0.99
Gastrointestinal bleed	4 (1.2)	2 (1.2)	2 (1.2)	0.99
Prolonged ventilation	11 (3.2)	5 (2.9)	6 (3.5)	0.76
30-day mortality	14 (4.1)	4 (2.3)	10 (5.8)	0.12
One-year mortality	26 (7.6)	14 (8.2)	12 (7.0)	0.74
Mortality during follow-up	148	66	82	
Reintervention during follow-up	15	5	10	

Values are shown as mean ± SD, median (interquartile) or *n* (%). Calculation based on complete observations. Summaries for unmatched data are presented in Supplementary Material, Table S7.

### Long-term survival

**Figure 1:**
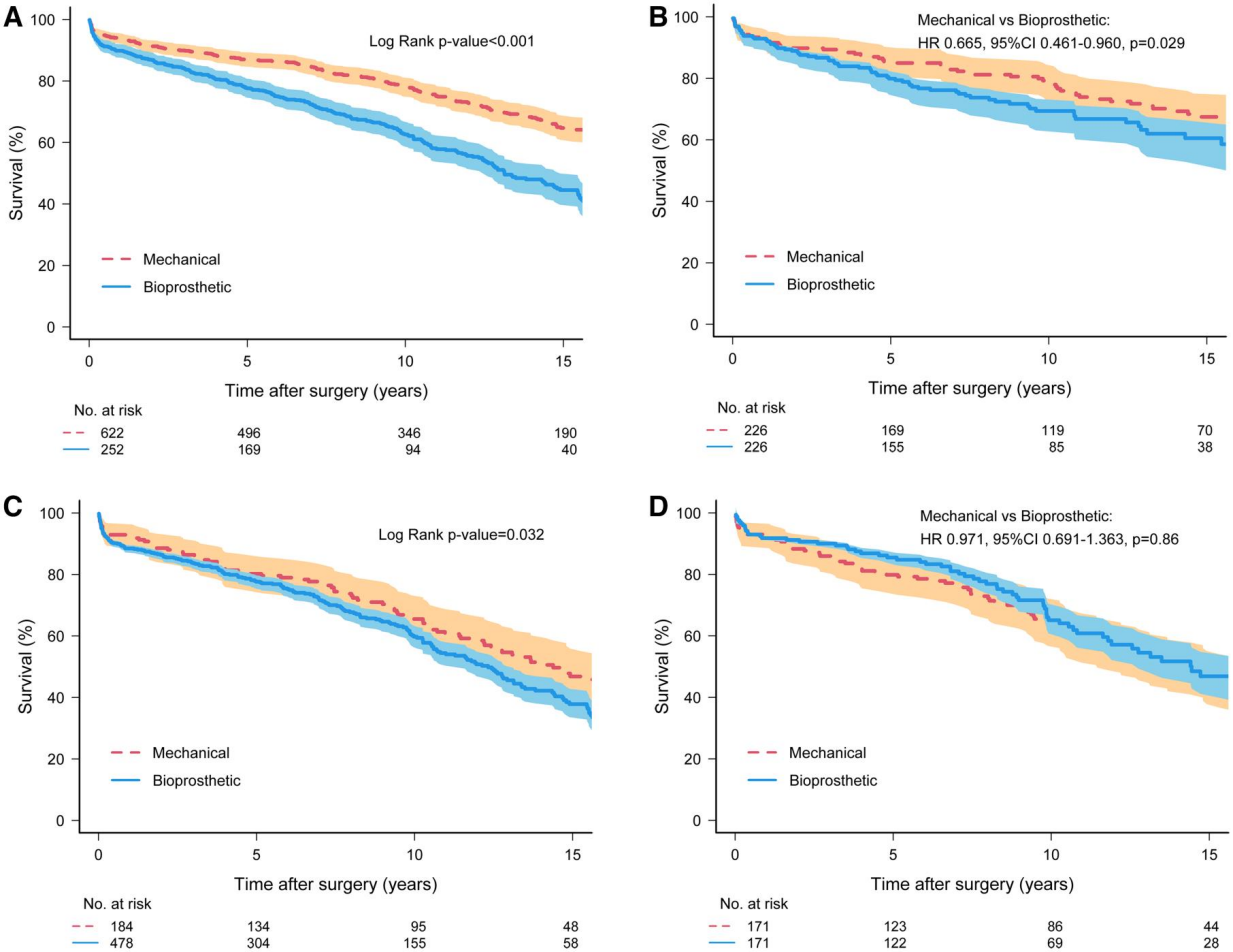
Kaplan–Meier curves of survival after mitral valve replacement. (**A**) Patients <65 years, unmatched; (**B**) patients <65 years, matched; (**C**) patients 65–75 years, unmatched; (**D**) patients 65–75 years, matched. 95% confidence limits are shown via shading. HR: hazard ratio.

**Figure 2:**
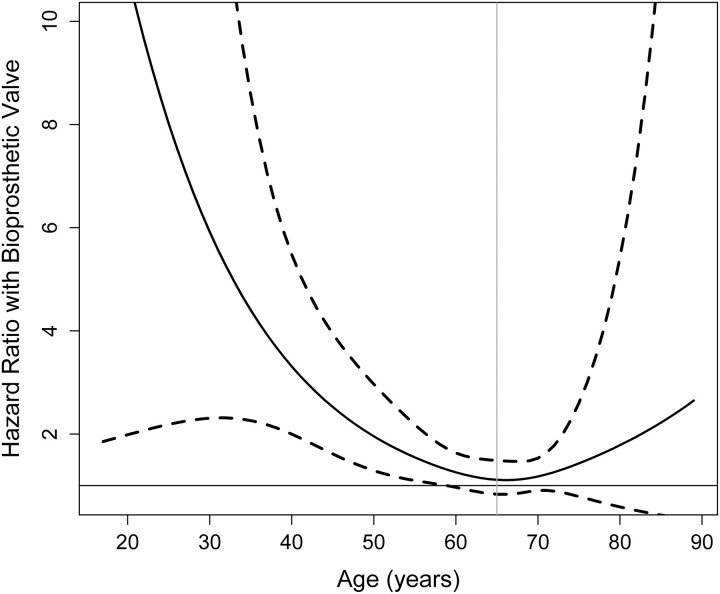
Estimated hazard ratios of bioprosthetic (versus mechanical) mitral valve replacement as a function of patient age. 95% Confidence intervals were denoted with hashed lines.

In matched patients aged <65 years, 10-year survival was significantly higher with mechanical valves than with bioprostheses [78.2 ± 2.8% vs 69.8 ± 3.4%, hazard ratio (HR) 0.665, 95% CI: 0.461–0.960, *P* = 0.029] (Fig. 1B; unmatched Fig. 1A). This survival benefit of mechanical MVR disappeared in matched patients aged 65–75 years, with similar 10-year survival rates between valve types (64.6 ± 3.8% vs 60.8 ± 4.1%, HR 0.971, 95% CI: 0.691–1.363, *P* = 0.86) (Fig. 1D; unmatched Fig. 1C). When age was examined as a continuous variable, the relative survival benefit associated with mechanical MVR persisted until ∼65 years of age based on HR (Fig. 2). The survival benefit of mechanical valves increased dramatically in patients of younger than 50–55 years. In contrast, bioprosthetic valves showed a trend towards survival benefit in patients 75 years or older (Fig. 2). Survival was also assessed using inverse probability of treatment weighting, which demonstrated similar results to the propensity-score matched comparison.

### Freedom from reintervention

**Figure 3:**
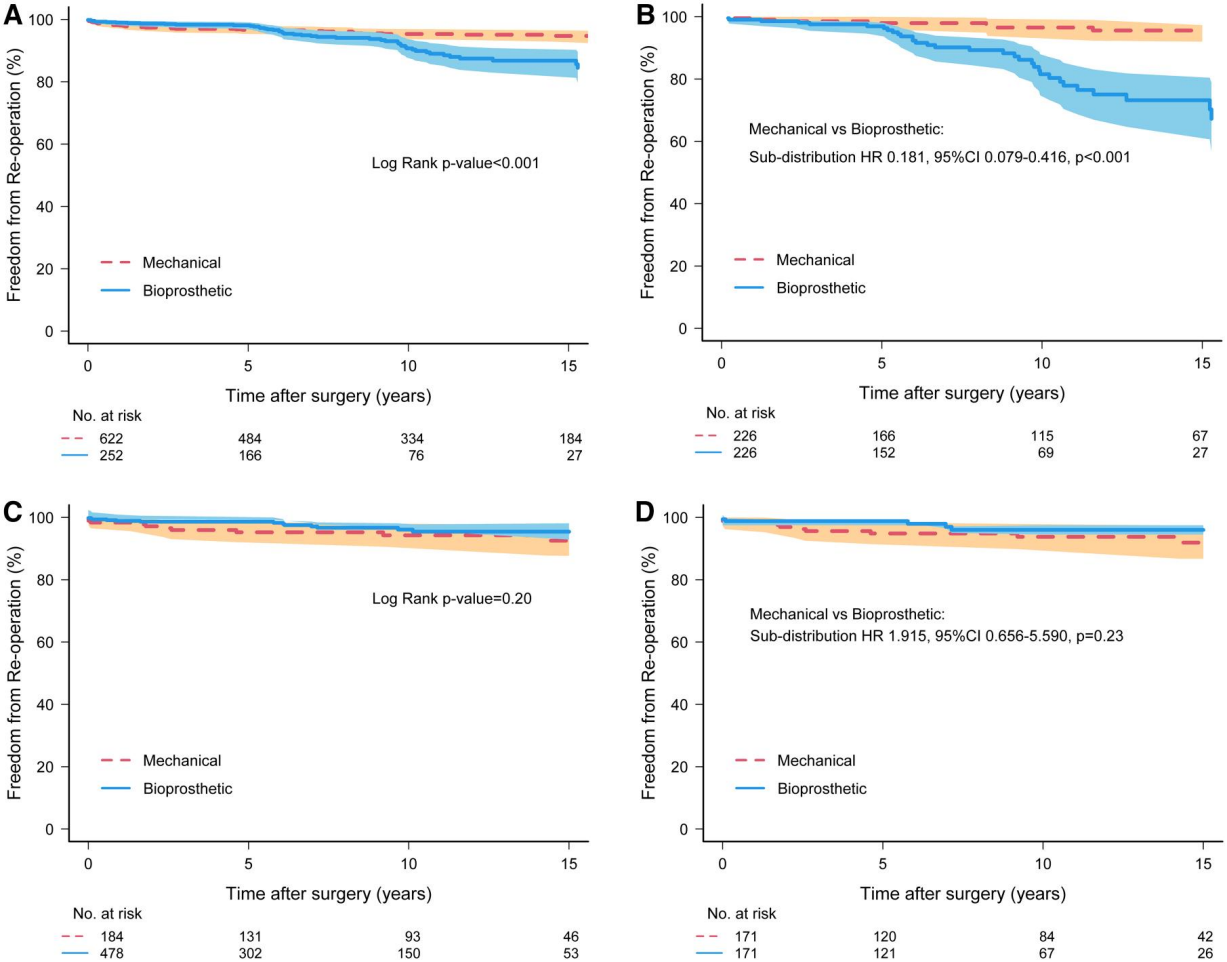
Kaplan–Meier curves of freedom from reintervention after mitral valve replacement. (**A**) patients <65 years, unmatched; (**B**) patients <65 years, matched; (**C**) patients 65–75 years, unmatched; (**D**) patients 65–75 years, matched. 95% Confidence limits are shown via shading. HR: hazard ratio.

In matched patients <65 years, 10-year freedom from reintervention was significantly higher following mechanical versus bioprosthetic MVR (96.2 ± 1.3% vs 81.3 ± 3.6%, sub-distribution HR 0.181, 95% CI: 0.079–0.416, *P* < 0.001) (Fig. 3B; unmatched Fig. 3A). This reintervention benefit of mechanical MVR disappeared in matched patients aged 65–75 years, with similar 10-year reintervention rates between valve types (94.0 ± 2.0% vs 97.2 ± 1.5%, sub-distribution HR 1.915, 95% CI: 0.656–5.590, *P* = 0.23) (Fig. 3D; unmatched Fig. 3C).

## DISCUSSION

In this multicentre, propensity-matched comparison of isolated mechanical versus bioprosthetic MVR, mechanical valves not only provided significant long-term survival benefit, but also significantly reduced valve reintervention in patients aged <65 years. However, the benefit of mechanical valves disappeared in patients aged 65–75 years.

Early postoperative complications, namely, stroke, gastrointestinal bleed and arrhythmia requiring permanent pacemaker insertion were similar between patients receiving isolated mechanical and bioprosthetic MVR. For patients aged <65 years, acute renal failure requiring dialysis occurred more frequently with bioprosthetic versus mechanical valves; this difference was not found in patients aged 65–75 years. Importantly, this difference did not translate to a difference in 30-day mortality between valve types.

In our study, we only included patients undergoing MVR without concomitant cardiac procedure besides arrhythmia procedures, which achieves the best possible balance between matched groups with selection biases minimized. When assessing clinical outcomes following MVR, concomitant coronary artery or valvular diseases and concomitant cardiac procedures may play a significant role in long-term outcomes. Propensity matching balances the number of concomitant procedures but would not appropriately balance the severity of coronary artery or valvular diseases between matched study groups. Many studies comparing bioprostheses and mechanical protheses in the mitral position have included sizeable proportions of patients receiving concomitant procedures, most commonly coronary artery bypass grafting, tricuspid valve repair or replacement or aortic valve replacement. In the non-propensity-matched studies of Goldstone *et al.* and Cetinkaya *et al.*, ∼35% of patients had concomitant coronary artery bypass grafting [10], and 20% of patients had concomitant tricuspid valve repair [6]. Even contemporary propensity-matched studies comparing mechanical and bioprosthetic MVR have included concomitant procedures in 15–85% of study cohorts [4, 5, 7, 8, 9, 11]. Inclusion of concomitant procedures may influence operative morbidity and mortality, as demonstrated by validated risk score calculators as well as previous studies from international databases [15, 16]. The uniqueness of this study is excluding any concomitant procedures except arrhythmic procedures (left atrial appendage resection/closure, pulmonary vein ablation or ablation Maze) to isolate the effect of prosthesis choice on long-term survival and freedom from reintervention.

Regarding long-term survival, we report a significant survival advantage for patients <65 years who received an isolated mechanical mitral valve, which became apparent at ∼5 years after operation and expanded in effect size to 8.4% (78.2% mechanical vs 69.8% bioprosthetic) at 10 years. However, in patients aged 65–75 years, this survival benefit was not observed. Our findings are consistent with other reports in which the survival benefit of mechanical over bioprosthetic MVR is shown in patients up to 65–69 years [7–11]. Two recent meta-analyses by Yanagawa *et al.* [17] and Yu *et al.* [18] also demonstrate survival benefit in patients <70 years but did not assess the 65–75 years cohort specifically. However, no difference in long-term survival was reported by Chikwe *et al.* [3], Bernard *et al.* [5] or Yu and Wang [4], all of which included patients in the 65–70 years age group in their analyses. Age-related subgroup analyses in these studies also did not demonstrate survival advantage of mechanical MVR. Of note, the only study to date which excluded concomitant procedures in a propensity-matched population was that of Chikwe *et al.* [3], which demonstrated no mortality benefit in any of the 10-year age subgroup analyses, contradictory to our present results. The main difference between the present study and that of Chikwe *et al.* is the age grouping: <65 years in our study versus 50–69 years in Chikwe’s study. In addition, 32 baseline factors were used in propensity matching in our study compared to 19 baseline factors.

Freedom from reintervention was significantly greater with use of mechanical prostheses compared to bioprostheses in isolated MVR in patients of <65 years in our study, consistent with many other studies in patients aged 50–70 years [3, 8–11]. Conversely, certain studies have demonstrated no difference in reintervention rates between bioprostheses and mechanical prostheses [4, 5, 7]. The 2 aforementioned meta-analyses both demonstrate significantly lower rates of reintervention in mechanical MVR versus bioprosthetic MVR, with HRs between 0.3 and 0.4 and absolute rates of reintervention similar to our study [17, 18].

Rates of other in-hospital complications, namely, stroke (∼2%), gastrointestinal bleeding (∼1%) and renal failure (∼4%) were similar between mechanical and bioprosthetic MVR in our study. These complication rates all appear on the mid-to-lower end of reported literature [3, 6, 7, 10, 11, 19]. Rates of postoperative arrhythmia requiring permanent pacemaker insertion were not commonly reported, though our rate (∼2%) appears lower than recent studies [6].

One of the primary drivers of bioprosthetic valve replacement is avoidance of oral anticoagulation. Unfortunately, our registry database did not contain long-term stroke and bleeding information. Nonetheless, increased risk of bleeding in patients receiving mechanical versus bioprosthetic valves is well established in previous literature [17, 18]. In this study, the vast majority of patients included received state-of-the-art mitral bioprostheses (Edwards Magna, Medtronic Mosaic and St Jude Medical Epic constituted over 95% over bioprostheses implanted). Previous literature included larger proportions of older bioprostheses [11] or did not specify bioprosthesis type [3, 7]. Bioprosthesis type represents an important data point to consider in discussions of mechanical versus bioprosthetic valve replacement in the mitral position and otherwise.

### Limitations

This study is associated with limitations inherent in any retrospective study. First, this cohort is from the provincial database of British Columbia, in which details of complications and anticoagulation after discharge are not documented. Therefore, long-term complications (particularly anticoagulation-related bleeding) were not available. However, this study focused on the impact of mechanical versus bioprosthetic valves on the long-term survival in propensity-matched patients with isolated MV surgery—increased risk of bleeding with anticoagulation has been well documented in previous publications [19]. Secondarily, detail on chordal preservation in MVR was not well documented in the registry and thus cannot be considered in the analysis. Furthermore, although the presence of concomitant atrial fibrillation surgery was recorded, details regarding specific operative management of atrial fibrillation were heterogeneously and sparsely captured in our database. Finally, causes of death were not available to us.

## CONCLUSION

Our study, which focused on patients receiving MVR with no concomitant coronary, valvular or aortic surgery, demonstrates that mechanical valves provide a significant long-term survival benefit and significantly reduced valve reintervention rate in patients <65 years; however, these benefits disappear in patients aged 65–75 years. Our findings suggest mechanical valves should be considered in patients <65 years and provide strong support to recommendations of current guidelines. In patients aged 65–75 years, bioprosthetic valves appear a better choice given no need for lifelong anticoagulation.

## Supplementary Material

ezae245_Supplementary_Data

## Data Availability

The data underlying this study will be shared upon request to the corresponding author.

## ABBREVIATIONS

HR: hazard ratio
MVR: Mitral valve replacement
NYHA: New York Heart Association
