# Supplementary material for: Long-term outcomes of isolated mechanical versus bioprosthetic mitral valve replacement in different age groups of propensity-matched patients
Source: Eur J Cardiothorac Surg. 2024 Jun 27;66(1):ezae245. doi: 10.1093/ejcts/ezae245 (PMC11223803; doi:10.1093/ejcts/ezae245)
Supplement: ezae245_Supplementary_Data [file ezae245_supplementary_data.docx]

**Supplementary Material**

**Table 1. Cohort by age groups and valve types**

| **Variable** | **All** | **Bioprosthetic** | **Bioprosthetic** | **Mechanical** | **Mechanical** |
| --- | --- | --- | --- | --- | --- |
|  | **n=1536** | **n=730** | **Matched** | **n=806** | **Matched** |
| Age < 65 yrs | 874 | **252** | **226**** | 622 | 171 |
| Age [65, 75) yrs | 662 | 478 | 226 | **184** | **171**** |

| **Table 2. Baseline characteristics before propensity score matching (entire cohort)** | | | | | |
| --- | --- | --- | --- | --- | --- |
| **Variable** | **All**  **n=1536** | **Tissue**  **n=730** | **Mechanical**  **n=806** | **P value** | **SMD** |
| Surgery age (yrs) | 59.8 ± 12.1 | 64.6 ± 11.3 | 55.5 ± 11.2 | <0.001 | -81.4 |
| Sex (Male) | 661 (43.0) | 320 (43.8) | 341 (42.3) | 0.55 | -3.1 |
| BMI (kg/m^2^ ) | 26.7 ± 6.1 | 26.4 ± 6.0 | 27.0 ± 6.1 | 0.07 | 9.2 |
| Creatinine (μmol/L) | 107.1 ± 95.3 | 106.4 ± 85.9 | 107.8 ± 103.2 | 0.79 | 1.4 |
| Hemoglobin (g/L) | 127.6 ± 19.7 | 124.9 ± 19.9 | 129.9 ± 19.3 | <0.001 | 25.5 |
| CCS Class |  |  |  | 0.010 | -13.3 |
| None | 1240 (80.7) | 575 (78.8) | 665 (82.5) |  |  |
| Class 1 or 2 | 176 (11.5) | 82 (11.2) | 94 (11.7) |  |  |
| Class 3 or 4 | 120 (7.8) | 73 (10.0) | 47 (5.8) |  |  |
| NYHA Class |  |  |  | <0.001 | -26.4 |
| None or Class I | 132 (8.6) | 54 (7.4) | 78 (9.7) |  |  |
| Class II | 376 (24.5) | 152 (20.8) | 224 (27.8) |  |  |
| Class III | 712 (46.4) | 331 (45.3) | 381 (47.3) |  |  |
| Class IV | 316 (20.6) | 193 (26.4) | 123 (15.3) |  |  |
| Ejection fraction (≤50%) | 588 (38.3) | 282 (38.6) | 306 (38.0) | 0.79 | 1.4 |
| Emergency or priority I for surgery | 273 (17.8) | 156 (21.4) | 117 (14.5) | <0.001 | -17.9 |
| Prior MI | 151 (9.8) | 91 (12.5) | 60 (7.4) | <0.001 | -16.8 |
| CHF | 1183 (77.0) | 573 (78.5) | 610 (75.7) | 0.19 | -6.7 |
| Active endocarditis | 105 (6.8) | 61 (8.4) | 44 (5.5) | 0.025 | -11.4 |
| Hypertension | 741 (48.2) | 389 (53.3) | 352 (43.7) | <.001 | -19.3 |
| Pulmonary hypertension | 880 (57.3) | 440 (60.3) | 440 (54.6) | 0.025 | -11.5 |
| Family history of CAD | 208 (13.5) | 120 (16.4) | 88 (10.9) | 0.002 | -16.1 |
| Dyslipidemia | 524 (34.1) | 284 (38.9) | 240 (29.8) | <0.001 | -19.3 |
| Pre-op arrhythmia | 591 (38.5) | 273 (37.4) | 318 (39.5) | 0.41 | 4.2 |
| Pre-op IABP | 11 (0.7) | 7 (1.0) | 4 (0.5) | 0.37 | -5.4 |
| PVD | 102 (6.6) | 64 (8.8) | 38 (4.7) | 0.001 | -16.2 |
| COPD | 368 (24.0) | 203 (27.8) | 165 (20.5) | <0.001 | -17.2 |
| Pre-op ventilation/Intubation | 49 (3.2) | 29 (4.0) | 20 (2.5) | 0.10 | -8.4 |
| Stroke/TIA | 279 (18.2) | 137 (18.8) | 142 (17.6) | 0.56 | -3.0 |
| Renal failure - acute. | 66 (4.3) | 42 (5.8) | 24 (3.0) | 0.007 | -13.6 |
| Renal failure - chronic | 100 (6.5) | 61 (8.4) | 39 (4.8) | 0.005 | -14.2 |
| Dialysis | 52 (3.4) | 24 (3.3) | 28 (3.5) | 0.84 | 1.0 |
| Diabetes | 238 (15.5) | 130 (17.8) | 108 (13.4) | 0.017 | -12.2 |
| Gastrointestinal bleed | 86 (5.6) | 56 (7.7) | 30 (3.7) | <0.001 | -17.1 |
| Malignant disease controlled < 5 years | 65 (4.2) | 44 (6.0) | 21 (2.6) | <0.001 | -16.9 |
| Smoking history - smoker or d/c < 1 mo | 174 (11.3) | 87 (11.9) | 87 (10.8) | 0.49 | -3.5 |
| History of drug abuse | 55 (3.6) | 31 (4.2) | 24 (3.0) | 0.18 | -6.8 |
| PCI | 81 (5.3) | 40 (5.5) | 41 (5.1) | 0.73 | -1.8 |
| Previous open heart surgery | 159 (10.4) | 86 (11.8) | 73 (9.1) | 0.08 | -8.9 |
| Values shown as mean ± SD, or n (%). *SMD*, standardized mean difference.  *BMI,* Body mass index; *CCS,* Canadian Cardiovascular Society; *NYHA*, New York Heart Association; *MI,* Myocardial infarction; *CHF,* Congestive heart failure; *CAD*, coronary artery disease; *IABP,* Intra-aortic balloon pump; *PVD*, Peripheral vascular disease; *COPD*, Chronic obstructive pulmonary disease; *TIA*, transient ischemic attack; *GI,* Gastrointestinal; *PCI*, Percutaneous Coronary Intervention. | | | | | |

| **Table 3. In-hospital and out-of-hospital outcomes before propensity score matching (entire cohort)** | | | | |
| --- | --- | --- | --- | --- |
| **Variable** | **All**  **n=1536** | **Tissue**  **n=730** | **Mechanical**  **n=806** | **P value** |
| Pump time (min) | 127.0 (96.0,172.0) | 130.0 (96.0,173.0) | 125.0 (95.0,169.0) | 0.26 |
| Cross clamp time (min) | 98.0 (73.0,136.0) | 100.0 (74.0,137.0) | 98.0 (72.0,135.0) | 0.32 |
| Creatinine-post (μmol/L) | 98.4 ± 83.4 | 96.4 ± 65.6 | 100.2 ± 96.6 | 0.40 |
| Prosthetic valve endocarditis | 1 (0.1) | 1 (0.1) | 0 (0.0) | -- |
| Insertion of permanent pacemaker | 20 (1.3) | 11 (1.5) | 9 (1.1) | 0.50 |
| Post-op hemorrhage/tamponade | 44 (2.9) | 18 (2.5) | 26 (3.2) | 0.37 |
| Arrhythmia - cardiac arrest | 20 (1.3) | 12 (1.6) | 8 (1.0) | 0.27 |
| Arrhythmia - atrial | 646 (42.1) | 316 (43.3) | 330 (40.9) | 0.35 |
| Arrhythmia - heart block | 76 (4.9) | 38 (5.2) | 38 (4.7) | 0.66 |
| Valvular thromboembolism/thrombosis | 0 (0.0) | 0 (0.0) | 0 (0.0) | -- |
| Inotropes > 24 hours | 84 (5.5) | 41 (5.6) | 43 (5.3) | 0.81 |
| Stroke | 27 (1.8) | 13 (1.8) | 14 (1.7) | 0.95 |
| Acute renal failure requiring dialysis | 66 (4.3) | 40 (5.5) | 26 (3.2) | 0.030 |
| Acute failure without dialysis | 58 (3.8) | 34 (4.7) | 24 (3.0) | 0.09 |
| Gastrointestinal bleed | 16 (1.0) | 10 (1.4) | 6 (0.7) | 0.23 |
| Prolonged ventilation | 50 (3.3) | 26 (3.6) | 24 (3.0) | 0.52 |
| \| 30-day mortality \| 38 (4.3) \| 17 (3.8) \| 21 (4.7) \| 0.52 \| \| --- \| --- \| --- \| --- \| --- \| | 64 (4.2) | 34 (4.7) | 30 (3.7) | 0.36 |
| One year mortality | 125 (8.1) | 75 (10.3) | 50 (6.2) | 0.004 |
| Mortality during follow-up | 536 (34.9) | 301 (41.2) | 235 (29.2) | -- |
| Reintervention during follow-up | 83 (5.4) | 48 (6.6) | 35 (4.3) | -- |
| Values shown as mean ± SD, or median (interquartile), or n (%). Calculation based on complete observations. | | | | |

| **Table 4. Baseline characteristics before propensity score matching (aged < 65 years)** | | | | | |
| --- | --- | --- | --- | --- | --- |
| **Variable** | **All**  **n=874** | **Tissue**  **n=252** | **Mechanical**  **n=622** | **P value** | **SMD** |
| Surgery age (yrs) | 51.8 ± 10.1 | 52.6 ± 11.4 | 51.6 ± 9.6 | 0.19 | -9.5 |
| Sex (Male) | 392 (44.9) | 123 (48.8) | 269 (43.2) | 0.13 | -11.2 |
| BMI (kg/m^2^) | 26.6 ± 6.2 | 25.3 ± 5.7 | 27.1 ± 6.4 | <0.001 | 30.4 |
| Creatinine (μmol/L) | 108.9 ± 108.9 | 110.3 ± 107.3 | 108.4 ± 109.7 | 0.81 | -1.8 |
| Hemoglobin (g/L) | 127.4 ± 21.2 | 122.6 ± 23.0 | 129.4 ± 20.2 | <0.001 | 31.2 |
| CCS Class |  |  |  | 0.25 | 13.8 |
| None | 721 (82.5) | 210 (83.3) | 511 (82.2) |  |  |
| Class 1 or 2 | 96 (11.0) | 22 (8.7) | 74 (11.9) |  |  |
| Class 3 or 4 | 57 (6.5) | 20 (7.9) | 37 (5.9) |  |  |
| NYHA Class |  |  |  | <0.001 | 45.2 |
| None or Class I | 86 (9.8) | 22 (8.7) | 64 (10.3) |  |  |
| Class II | 246 (28.1) | 61 (24.2) | 185 (29.7) |  |  |
| Class III | 377 (43.1) | 98 (38.9) | 279 (44.9) |  |  |
| Class IV | 165 (18.9) | 71 (28.2) | 94 (15.1) |  |  |
| Ejection fraction (≤50%) | 318 (36.4) | 88 (34.9) | 230 (37.0) | 0.57 | -4.3 |
| Emergency or priority I for surgery | 160 (18.3) | 69 (27.4) | 91 (14.6) | <.001 | -31.7 |
| Prior MI | 75 (8.6) | 35 (13.9) | 40 (6.4) | <.001 | -24.9 |
| CHF | 650 (74.4) | 195 (77.4) | 455 (73.2) | 0.19 | -9.8 |
| Active endocarditis | 80 (9.2) | 40 (15.9) | 40 (6.4) | <.001 | -30.3 |
| Hypertension | 343 (39.2) | 98 (38.9) | 245 (39.4) | 0.89 | 1.0 |
| Pulmonary hypertension | 476 (54.5) | 146 (57.9) | 330 (53.1) | 0.19 | -9.8 |
| Family history of CAD | 113 (12.9) | 46 (18.3) | 67 (10.8) | 0.003 | -21.4 |
| Dyslipidemia | 244 (27.9) | 74 (29.4) | 170 (27.3) | 0.54 | -4.5 |
| Pre-op arrhythmia | 280 (32.0) | 67 (26.6) | 213 (34.2) | 0.028 | 16.7 |
| Pre-op IABP | 7 (0.8) | 4 (1.6) | 3 (0.5) | 0.10 | -10.9 |
| PVD | 51 (5.8) | 21 (8.3) | 30 (4.8) | 0.045 | -14.2 |
| COPD | 202 (23.1) | 74 (29.4) | 128 (20.6) | 0.005 | -20.4 |
| Pre-op ventilation/Intubation | 39 (4.5) | 20 (7.9) | 19 (3.1) | 0.002 | -21.6 |
| CVA/TIA | 156 (17.8) | 49 (19.4) | 107 (17.2) | 0.43 | -5.8 |
| Renal failure - acute. | 38 (4.3) | 19 (7.5) | 19 (3.1) | 0.003 | -20.1 |
| Renal failure - chronic | 47 (5.4) | 21 (8.3) | 26 (4.2) | 0.014 | -17.2 |
| Dialysis | 36 (4.1) | 12 (4.8) | 24 (3.9) | 0.54 | -4.5 |
| Diabetes | 107 (12.2) | 31 (12.3) | 76 (12.2) | 0.97 | -0.3 |
| Peptic ulcer, GI bleed history | 48 (5.5) | 23 (9.1) | 25 (4.0) | 0.003 | -20.7 |
| Malignant disease controlled < 5 years | 17 (1.9) | 8 (3.2) | 9 (1.4) | 0.09 | -11.5 |
| Smoking history - smoker or d/c < 1 mo | 136 (15.6) | 58 (23.0) | 78 (12.5) | <.001 | -27.7 |
| History of drug abuse | 53 (6.1) | 29 (11.5) | 24 (3.9) | <.001 | -29.0 |
| PCI | 40 (4.6) | 13 (5.2) | 27 (4.3) | 0.60 | -3.9 |
| Previous open heart surgery | 83 (9.5) | 33 (13.1) | 50 (8.0) | 0.021 | -16.5 |
| Values shown as mean ± sd, or n(%). Abbreviations referred to Supplementary Table 2. | | | | | |

| **Table 5. Baseline characteristics before propensity score matching (aged 65-75 years)** | | | | | |  |
| --- | --- | --- | --- | --- | --- | --- |
| **Variable** | | **All**  **n=662** | **Tissue**  **n=478** | **Mechanical**  **n=184** | **P value** | **SMD** |
| Surgery age (yrs) | | 70.4 ± 3.2 | 71.0 ± 3.1 | 68.7 ± 2.7 | <0.001 | -77.2 |
| Sex (Male) | | 269 (40.6) | 197 (41.2) | 72 (39.1) | 0.63 | -4.2 |
| BMI (kg/m^2^ ) | | 27.0 ± 5.8 | 27.1 ± 6.1 | 26.7 ± 5.1 | 0.41 | -7.4 |
| Creatinine (μmol/L) | | 104.8 ± 73.7 | 104.4 ± 72.0 | 105.7 ± 77.9 | 0.84 | -1.7 |
| Hemoglobin (g/L) | | 127.7 ± 17.6 | 126.1 ± 17.9 | 131.8 ± 16.0 | <0.001 | 33.2 |
| CCS Class | |  |  |  | 0.06 | 21.0 |
| None | | 519 (78.4) | 365 (76.4) | 154 (83.7) |  |  |
| Class 1 or 2 | | 80 (12.1) | 60 (12.6) | 20 (10.9) |  |  |
| Class 3 or 4 | | 63 (9.5) | 53 (11.1) | 10 (5.4) |  |  |
| NYHA Class | |  |  |  | 0.07 | 32.3 |
| None or Class I | | 46 (6.9) | 32 (6.7) | 14 (7.6) |  |  |
| Class II | | 130 (19.6) | 91 (19) | 39 (21.2) |  |  |
| Class III | | 335 (50.6) | 233 (48.7) | 102 (55.4) |  |  |
| Class IV | | 151 (22.8) | 122 (25.5) | 29 (15.8) |  |  |
| Ejection fraction (≤50%) | | 270 (40.8) | 194 (40.6) | 76 (41.3) | 0.87 | -1.5 |
| Emergency or priority I for surgery | | 113 (17.1) | 87 (18.2) | 26 (14.1) | 0.21 | -11.1 |
| Prior MI | | 76 (11.5) | 56 (11.7) | 20 (10.9) | 0.76 | -2.7 |
| CHF | | 533 (80.5) | 378 (79.1) | 155 (84.2) | 0.13 | 13.4 |
| Active endocarditis | | 25 (3.8) | 21 (4.4) | 4 (2.2) | 0.18 | -12.5 |
| Hypertension | | 398 (60.1) | 291 (60.9) | 107 (58.2) | 0.52 | -5.6 |
| Pulmonary hypertension | | 404 (61.0) | 294 (61.5) | 110 (59.8) | 0.68 | -3.5 |
| Family history of CAD | | 95 (14.4) | 74 (15.5) | 21 (11.4) | 0.18 | -11.9 |
| Dyslipidemia | | 280 (42.3) | 210 (43.9) | 70 (38) | 0.17 | -12.0 |
| Pre-op arrhythmia | | 311 (47.0) | 206 (43.1) | 105 (57.1) | 0.001 | 28.2 |
| Pre-op IABP | | 4 (0.6) | 3 (0.6) | 1 (0.5) | 0.90 | -1.1 |
| PVD | | 51 (7.7) | 43 (9) | 8 (4.3) | 0.045 | -18.7 |
| COPD | | 166 (25.1) | 129 (27) | 37 (20.1) | 0.07 | -16.3 |
| Pre-op ventilation/Intubation | | 10 (1.5) | 9 (1.9) | 1 (0.5) | 0.21 | -12.3 |
| CVA/TIA | | 123 (18.6) | 88 (18.4) | 35 (19) | 0.86 | 1.6 |
| Renal failure - acute. | | 28 (4.2) | 23 (4.8) | 5 (2.7) | 0.23 | -11.0 |
| Renal failure - chronic | | 53 (8.0) | 40 (8.4) | 13 (7.1) | 0.58 | -4.9 |
| Dialysis | | 16 (2.4) | 12 (2.5) | 4 (2.2) | 0.80 | -2.2 |
| Diabetes | | 131 (19.8) | 99 (20.7) | 32 (17.4) | 0.34 | -8.5 |
| Peptic ulcer, GI bleed history | | 38 (5.7) | 33 (6.9) | 5 (2.7) | 0.038 | -19.7 |
| Malignant disease controlled < 5 years | | 48 (7.3) | 36 (7.5) | 12 (6.5) | 0.65 | -4.0 |
| Smoking history - smoker or d/c < 1 mo | | 38 (5.7) | 29 (6.1) | 9 (4.9) | 0.56 | -5.2 |
| History of drug abuse | | 2 (0.3) | 2 (0.4) | 0 (0.0) | 0.38 | -5.2 |
| PCI | | 41 (6.2) | 27 (5.6) | 14 (7.6) | 0.35 | 7.9 |
| Previous open heart surgery | | 76 (11.5) | 53 (11.1) | 23 (12.5) | 0.61 | 4.4 |
| Values shown as mean ± sd, or n(%). Abbreviations referred to Supplementary Table 2. | | | | | |  |

| **Table 6. In-hospital and out-of-hospital outcomes before propensity score matching (aged < 65 years)** | | | | |
| --- | --- | --- | --- | --- |
| **Variable** | **All**  **n=874** | **Tissue**  **n=252** | **Mechanical**  **n=622** | **P value** |
| Pump time (min) | 125.0 (94.0, 168.0) | 123.0 (91.0, 166.0) | 126.0 (95.0, 168.0) | 0.60 |
| Cross clamp time (min) | 97.0 (70.0, 133.0) | 95.0 (70.0, 131.0) | 98.0 (71.0, 134.0) | 0.58 |
| Creatinine-post (μmol/L) | 97.7 ± 85.7 | 94.4 ± 77.0 | 101.2 ± 93.9 | 0.34 |
| Prosthetic valve endocarditis | 0 (0.0) | 0 (0.0) | 0 (0.0) | -- |
| Insertion of permanent pacemaker | 10 (1.1) | 3 (1.2) | 7 (1.1) | 0.94 |
| Post-op hemorrhage/tamponade | 24 (2.7) | 6 (2.4) | 18 (2.9) | 0.67 |
| Arrhythmia - cardiac arrest | 10 (1.1) | 4 (1.6) | 6 (1.0) | 0.43 |
| Arrhythmia - atrial | 340 (38.9) | 87 (34.5) | 253 (40.7) | 0.09 |
| Arrhythmia - heart block | 42 (4.8) | 13 (5.2) | 29 (4.7) | 0.76 |
| Valvular thromboembolism/thrombosis | 0 (0.0) | 0 (0.0) | 0 (0.0) | -- |
| Inotropes > 24 hours | 46 (5.3) | 15 (6.0) | 31 (5.0) | 0.56 |
| Stroke | 12 (1.4) | 4 (1.6) | 8 (1.3) | 0.73 |
| Acute renal failure requiring dialysis | 38 (4.3) | 18 (7.1) | 20 (3.2) | 0.010 |
| Acute failure without dialysis | 23 (2.6) | 8 (3.2) | 15 (2.4) | 0.52 |
| Gastrointestinal bleed | 7 (0.8) | 3 (1.2) | 4 (0.6) | 0.41 |
| Prolonged ventilation | 28 (3.2) | 10 (4) | 18 (2.9) | 0.41 |
| 30-day mortality | 28 (3.2) | 8 (3.2) | 20 (3.2) | 0.99 |
| One year mortality | 57 (6.5) | 20 (7.9) | 37 (5.9) | 0.28 |
| Mortality during follow-up | 234 (26.8) | 86 (34.1) | 148 (23.8) | -- |
| Reintervention during follow-up | 60 (6.9) | 35 (13.9) | 25 (4) | -- |
| Values shown as mean ± SD, or median (interquartile) or n (%). Calculation based on complete observations. | | | | |

| **Table 7. In-hospital and out-of-hospital outcomes before propensity score matching (aged 65-75 years)** | | | | |
| --- | --- | --- | --- | --- |
| **Variable** | **All**  **n=662** | **Tissue**  **n=478** | **Mechanical**  **n=184** | **P value** |
| Pump time (min) | 130.0 (98.0, 175.0) | 132.0 (100.0, 176.0) | 125.0 (96.5, 171.0) | 0.27 |
| Cross clamp time (min) | 100.0 (76.0, 140.0) | 102.5 (78.0, 140.0) | 95.0 (73.0, 142.0) | 0.35 |
| Creatinine-post (μmol/L) | 99.2 ± 72.4 | 98.3 ± 60.6 | 101.7 ± 97.2 | 0.62 |
| Prosthetic valve endocarditis | 1 (0.2) | 1 (0.2) | 0 (0.0) | 0.54 |
| Insertion of permanent pacemaker | 10 (1.5) | 8 (1.7) | 2 (1.1) | 0.58 |
| Post-op hemorrhage/tamponade | 20 (3.0) | 12 (2.5) | 8 (4.3) | 0.22 |
| Arrhythmia - cardiac arrest | 10 (1.5) | 8 (1.7) | 2 (1.1) | 0.58 |
| Arrhythmia - atrial | 306 (46.2) | 229 (47.9) | 77 (41.8) | 0.16 |
| Arrhythmia - heart block | 34 (5.1) | 25 (5.2) | 9 (4.9) | 0.86 |
| Valvular thromboembolism/thrombosis | 0 (0.0) | 0 (0.0) | 0 (0.0) | -- |
| Inotropes > 24 hours | 38 (5.7) | 26 (5.4) | 12 (6.5) | 0.59 |
| Stroke | 15 (2.3) | 9 (1.9) | 6 (3.3) | 0.29 |
| Acute renal failure requiring dialysis | 28 (4.2) | 22 (4.6) | 6 (3.3) | 0.44 |
| Acute failure without dialysis | 35 (5.3) | 26 (5.4) | 9 (4.9) | 0.78 |
| Gastrointestinal bleed | 9 (1.4) | 7 (1.5) | 2 (1.1) | 0.71 |
| Prolonged ventilation | 22 (3.3) | 16 (3.3) | 6 (3.3) | 0.96 |
| 30-day mortality | 36 (5.4) | 26 (5.4) | 10 (5.4) | 0.99 |
| One year mortality | 68 (10.3) | 55 (11.5) | 13 (7.1) | 0.09 |
| Mortality during follow-up | 302 (45.6) | 215 (45.0) | 87 (47.3) | -- |
| Reintervention during follow-up | 23 (3.5) | 13 (2.7) | 10 (5.4) | -- |
| Values shown as mean ± SD, or median (interquartile) or n (%). Calculation based on complete observations. | | | | |
